# Hernie de Spiegel: a propos d’un cas

**Published:** 2010-02-08

**Authors:** Karim Ibn Majdoub Hassani, Fatimzohra Zahid, Hicham Anoune, Imane Toughrai, Said Ait Laalim, Khalid Mazaz

**Affiliations:** 1 Service de chirurgie B, CHU Hassan II FES, Maroc

**Keywords:** Hernie de Spiegel, Maroc, Chirurgie abdominale

## Abstract

**Introduction:**

La hernie de Spiegel ou hernie ventrale latérale est une déhiscence inhabituelle apparaissant sur la ligne ou fascia semi-lunaire de Spiegel. C’est une entité clinique rare, représente 0.10 à 1% des hernies. Aussi, nous a-t-il paru opportun de rapporter ce cas colligé dans le service de chirurgie B du CHU Hassan II de Fès.

**Patient et observation:**

Nous rapportons l’observation d’une patiente âgée de 60ans, sans antécédent particulier qui présentais une tuméfaction para ombilicale gauche augmentant progressivement de volume, Une hernie de Spiegel a été suspectée à l’examen clinique, et le diagnostic d’éventration antérolatérale gauche a été retenu à la tomodensitométrie abdominale. Une cure de la hernie par plaque de prolène a été réalisée et les suites opératoires étaient simples.

**Conclusion:**

La hernie de Spiegel est une affection rare, son diagnostic clinique peut être difficile. Elle est asymptomatique dans 90% des cas et Son diagnostic positif est radiologique. Le risque d’étranglement non négligeable impose un traitement chirurgical une fois le diagnostic est confirmé.

## Introduction

La réputation de la rareté de la hernie de Spiegel est connue, elle constitue 0,1 à 1% des hernies de la paroi abdominale [1]. C’est une hernie généralement localisée en para et sous ombilicale, située dans la ligne semi lunaire (aponévrose du petit oblique et du transverse). Elle peut contenir de l’épiploon, du grêle et du colon et son contenu peut être réductible ou non. Son diagnostic positif est radiologique et la chirurgie constitue son unique traitement. A la lumière d’une observation et d’une revue de la littérature, Le but de notre travail est de mettre en exergue les difficultés diagnostics et les différentes modalités thérapeutiques de cette pathologie.

## Patient et observation

**Figure 1:**
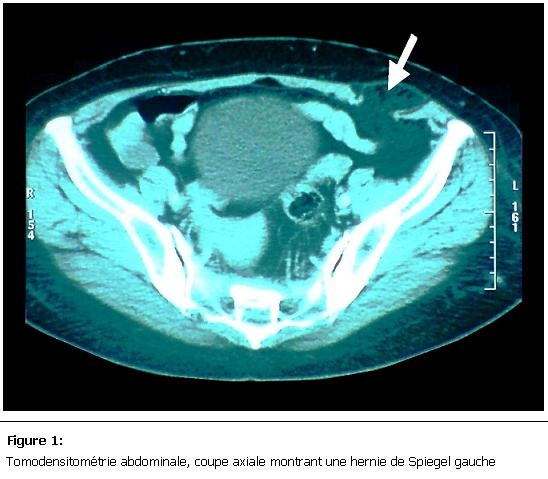
Tomodensitométrie abdominale, coupe axiale montrant une hernie de Spiegel gauche

Nous rapportons le cas d’une patiente âgée de 60 ans, mère de 4 enfants, obèse, présentant depuis 3ans une masse para rectale gauche en sous ombilicale, augmentant progressivement de volume, sans trouble de transit et sans altération de l’état générale. A l’examen il s’agissait d’une masse d’environ 12 cm de diamètre, molle, non douloureuse et non réductible, sans signe inflammatoire en regard, faisant évoquer en premier lieu une prolifération lipomateuse. L’échographie avait suspecté une hernie de spiegel, la confirmation diagnostic était obtenue par la TDM abdominale qui avait montré une déhiscence musculaire en sous ombilicale au niveau de la ligne semi lunaire de spiegel avec présence du sac herniaire contenant le grand épiploon (Figures 1 et 2).

**Figure 2:**
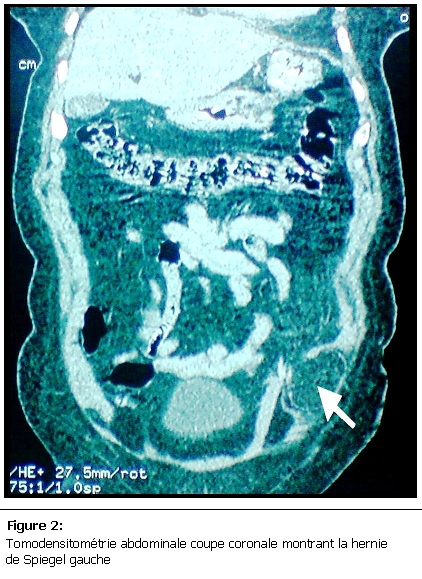
Tomodensitométrie abdominale coupe coronale montrant la hernie de Spiegel gauche

**Figure 3:**
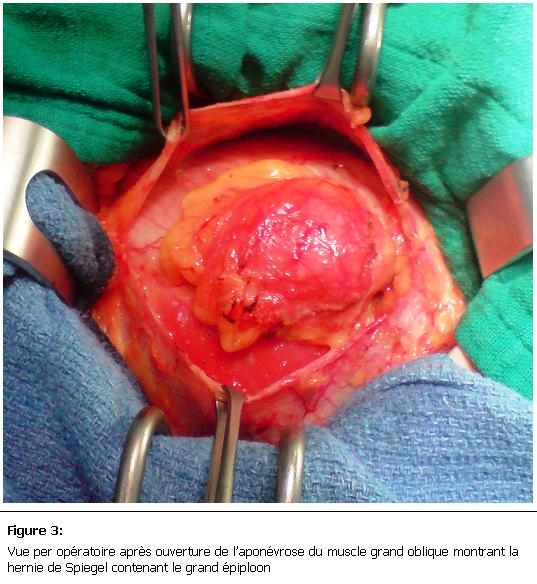
Vue per opératoire après ouverture de l’aponévrose du muscle grand oblique montrant la hernie de Spiegel contenant le grand épiploon

**Figure 4:**
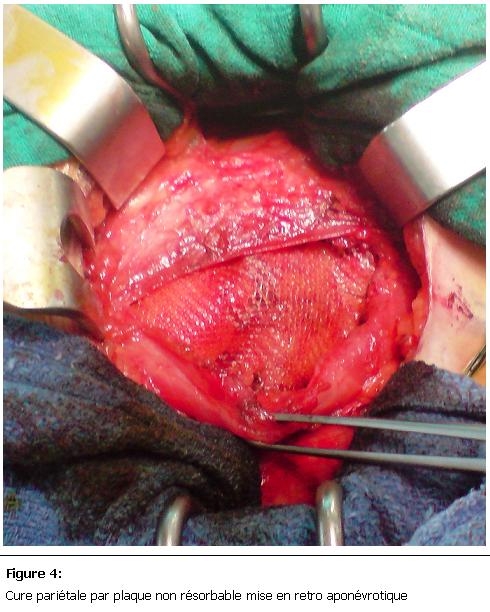
Cure pariétale par plaque non résorbable mise en retro aponévrotique

L’exploration chirurgicale avait retrouvé une hernie de spiegel contenant du grand épiploon viable (Figure 3), le traitement avait consisté en une réduction de la hernie avec une cure pariétale par plaque non résorbable (Figure 4). Les suites opératoires étaient simples.

## Discussion

La ligne semi lunaire à été décrite pour la première fois par Adriaan van der Spieghel en 1645 [2], elle correspond à la jonction au bord latéral externe des muscles droits de l'abdomen des aponévroses des muscles larges. C’est une pathologie rare mais non exceptionnelle (moins de 1000 publications dans le monde) [3]. Elle survient généralement après l’âge de 40 ans [4], elle est le plus souvent rencontrées au dessous du niveau ombilical par déhiscence de l’aponévrose du transverse et du muscle oblique interne qui paraissent plus faible au voisinage de la ligne arquée. Les principaux facteurs de risque sont l’obésité et la grossesse.

Le diagnostic clinique de l’hernie de spiegel est souvent rendu difficile par l’obésité, soit lorsque l’hernie est de petite taille elle est alors à peine palpable, soit en cas d’une hernie importante faisant saillie sous la peau elle peut être confondu avec un lipome comme dans le cas de notre observation ou avec une tumeur intra abdominale. Il est estimé qu’autour de 50% des patients ayant une hernie de spiegel n’avaient pas un diagnostic préopératoire correct [5].

La TDM abdominale reste l’examen clé pour la confirmation du diagnostique avec une grande sensibilité, elle permet de voir la déhiscence musculaire et le contenu du sac herniaire [6]. Comme toute hernie de la paroi ventro-latérale de l’abdomen le risque d’incarcération ou d’étranglement est important, et peut allez jusqu'à 32% [7], raison pour laquelle, le traitement chirurgical doit être instauré le plus rapidement possible. La cure pariétale par plaque prothétique est le traitement de choix [6], la voie laparoscopique en plus de son intérêt diagnostic avait prouvé son efficacité dans le traitement de ce type d’hernie [3, 8], la voie conventionnel est toujours de mise surtout dans les hernies de grosse taille.

## Conclusion

La hernie de spiegel est une affection rare de l’adulte jeune, le diagnostic clinique est parfois difficile, mais le scanner garde une grande sensibilité pour la confirmation du diagnostic. Le risque d’étranglement non négligeable impose un traitement chirurgical une fois le diagnostic est confirmé.

## Consentement

Les auteurs déclarent avoir reçu le consentement écrit de la patiente pour reporter ce cas.

## Conflits d’intérêts

Les auteurs déclarent n’avoir aucuns conflits d’intérêts.

## Contribution des auteurs

**KIM** a rédigé l’article, **FZZ** a contribué à la prise des photos, **HA** et **IT** ont contribué à la recherche bibliographique, les autres auteurs ont contribué à la prise en charge thérapeutique de la malade et à la rédaction de ce document.
